# Toll/Interleukin-1 receptor member ST2 exhibits higher soluble levels in type 2 diabetes, especially when accompanied with left ventricular diastolic dysfunction

**DOI:** 10.1186/1475-2840-10-101

**Published:** 2011-11-21

**Authors:** Evangelos Fousteris, Andreas Melidonis, George Panoutsopoulos, Konstantinos Tzirogiannis, Stefanos Foussas, Anastasios Theodosis-Georgilas, Stavros Tzerefos, Spiridon Matsagos, Eleni Boutati, Theofanis Economopoulos, George Dimitriadis, Sotirios Raptis

**Affiliations:** 1 Diabetes Center, "Tzanio" General Hospital of Piraeus, Greece; 2 Department of Nursing, Faculty of Human Movement and Quality of Life Sciences, University of Peloponnese, Greece; 3 Cardiology Department, Tzanio General Hospital of Piraeus, Greece; 4 Blood Bank Service, Tzanio General Hospital of Piraeus, Greece; 5 2nd Department of Internal Medicine, Research Institute & Diabetes Center, "Attikon" University General Hospital, Greece; 6 Hellenic National Diabetes Center, Athens, Greece

**Keywords:** Soluble ST2, BNP, hs-CRP, type 2 diabetes, diastolic dysfunction

## Abstract

**Background:**

Soluble ST2, a member of the of the Toll/IL-1 superfamily, is a novel biomarker with exceptional predictive value in heart failure and myocardial infarction- related mortality as well as in acute dyspneic states. Soluble ST2 is considered a decoy receptor of IL 33 that blocks the protective effects of the cytokine in atherosclerosis and cardiac remodeling. In the present study we investigated the differences in the levels of soluble ST2, BNP and hs-CRP between healthy controls and patients with type 2 diabetes with and without left ventricular diastolic dysfunction. A secondary aim was to investigate correlations between sST2 and other biomarkers of type 2 diabetes, such as HbA1c.

**Methods:**

158 volunteers were recruited and underwent a complete Doppler-echocardiographic evaluation of both systolic & diastolic cardiac function. All subjects with ejection fraction < 50% were excluded. The study population was divided in 4 groups as follows: A: 42 healthy controls, B: 18 subjects without diabetes with LVDD, C: 48 patients with type 2 diabetes without LVDD & D: 50 patients with type 2 diabetes & LVDD. ELISA technique was performed to measure sST2 levels. Statistical analysis was performed with Kruskal-Wallis & Mann-Whitney test (continuous variables), chi squared & Fischer exact test (discrete variables), Spearman coefficient (univariate analysis) and step-wise backward method (multivariate analysis).

**Results:**

Patients with type 2 diabetes with (p < 0.001) or without LVDD (p = 0.007) had higher serum ST2 levels compared to healthy controls, state found also for hs-CRP levels but not for the corresponding BNP levels (p = 0.213 & p = 0.207 respectively). Patients with type 2 diabetes & LVDD had higher serum ST2 in relation to diabetic patients without LVDD (p = 0.001). In multivariate analysis HbA1c positively and independently correlated with sST2 levels in both groups of patients with type 2 diabetes.

**Conclusions:**

Patients with type 2 diabetes exhibit higher sST2 levels compared to healthy controls. The presence of LVDD in patients with type 2 diabetes is associated with even higher sST2 levels. A significant correlation between glycemic control and sST2 levels was also revealed.

## Background

Soluble ST2, a member of the of the Toll/IL-1 superfamily, is a novel biomarker of myocardial mechanical stress with exceptional predictive value in heart failure and myocardial infarction- related mortality [1-7] as well as in acute dyspneic states [8-11]. Soluble ST2 is considered a decoy receptor of IL 33 (a member of IL-1 receptor family of cytokines) that blocks the protective effects of the cytokine in atherosclerosis, obesity and cardiac remodeling [3,4].

IL-33 is a cytokine with dual function, acting both as a traditional cytokine implicated in numerous inflammatory disorders and as a transcriptional factor [12].IL-33 is expressed in various tissues and in the heart and vascular tree and is considered to play a significant role in various cardiovascular disorders [13]. In relation to atherosclerosis, IL-33 is speculated to exert an anti-atherosclerotic effect by inducing a Th1-to-Th2 immune response [13], arresting foam cell formation [14], stimulating IL-5 production and oxidized low-density lipoprotein antibodies [15]. Downstream activation of NF-kB and the MAPK kinases by IL-33 also competitively inhibits excessive activation of these pathways by more potent activators, such as angiotensin II and phenylephrine, that are connected to increased ROS production [16]. The role of IL33/ST2 signaling pathway in the heart remains hugely unveiled today and according to current knowledge it is considered a paracrine cardioprotective pathway between cardiomyocytes and cardiac fibroblasts [16]. In an animal model of pressure overload, IL 33 treatment reduced cardiac hypertrophy and fibrosis and improved survival [16]. The same cytokine also exerts antiapoptotic effects through suppression of caspace-3 and increased expression of inhibitors of apoptosis [17].

In the immune system IL-33, through its receptor T1/ST2 (transmembrane form of ST2), exerts a pivotal role in Th2 responses, as well as in mast cell and eosinophil activation through activation of NF-kB and MAP Kinases [18-20]. Soluble ST2 levels have been found elevated in an array of human diseases including asthma, allergic airway inflammation, systemic lupus erythematosus, rheumatoid arthritis, idiopathic pulmonary fibrosis and sepsis. Several studies have also shown a beneficial effect after soluble ST2 administration in animal models of inflammatory disease [18]. These results have been attributed to the blockade of IL 33 actions with downstream suppression of NF-kB activation [18].

In relation to diabetes, IL33 exerted protective effects in an animal model of obese diabetic mice (ob/ob) reducing adiposity, fasting plasma glucose and improving glucose tolerance and insulin resistance [21].

In the present study we investigated the differences in the levels of soluble ST2, B-type natriuretic peptide (BNP) and high sensitivity C - reactive protein (hs-CRP) between healthy controls and patients with type 2 diabetes with and without left ventricular diastolic dysfunction (LVDD). A secondary aim was to investigate correlations between sST2 and other biomarkers of type 2 diabetes, such as glycosylated hemoglobin (HbA1c).

## Methods

This is a prospectively conducted study that took place in an urban general hospital in Piraeus, Greece from July 2009 to July 2010. The study protocol is in compliance with the Helsinki Declaration and was approved by the ethical committee of the hospital (5/10-04-2009). The diabetic population of our sample was recruited from the Diabetes Center of our hospital. People with type 2 diabetes were informed about the study protocol and were voluntarily recruited after consent. People without diabetes (controls) were chosen from the echocardiography unit of the cardiology department of the same hospital among patients without history of cardiac diseases, in whom the indication for echocardiography was "atypical chest pain". They were also voluntarily recruited after informed consent.

Subjects with impaired systolic function (ejection fraction of left ventricle < 50%) or history of coronary artery disease were excluded. Other exclusion criteria included history of: arrhythmias, cardiac valvular disorders, malignancies, autoimmune diseases and other inflammatory states, Cushing's syndrome and use of corticosteroids or thiazolinediones.

Subjects underwent a complete Doppler-echocardiographic evaluation. Echocardiography (and especially Tissue Doppler Imaging) was used to diagnose subjects with left ventricular systolic and/or diastolic dysfunction (according to the revised guidelines of A.C.C./A.H.A. 2009). Studies were performed on a Vivid 7 echocardiography machine (Vingmed, Norway). All measurements were made by a single experienced echocardiographer blinded to the diabetic status of the patients according to the ASE recommendations for chamber quantification 20051. The LV ejection fraction was estimated using the Simpson biplane method. From the mitral inflow profile, the E- and A-wave peak velocities and DT were measured. The E' velocity from the septal and lateral mitral valve annulus and the mean value were determined, and the respective E/E' ratios were derived. An E/E'septal ratio >15 was considered indicative of elevated LV filling pressure. Diastolic function was categorized using mitral inflow and Doppler Tissue Imaging parameters. **Grade 3 **diastolic dysfunction (restrictive) was defined as an E/A ratio >2 and/or DT <150 milliseconds; and **Grade 1 **dysfunction (impaired relaxation), as an E/A ratio <1 and/or a DT >220 milliseconds. **Grade 2 **dysfunction (pseudonormal) was diagnosed where the DT was between 150 and 220 milliseconds and the E/A ratio was between 1 and 2, but either the E/e'septal was >10 and/or the E/A ratio fell to <1 with Valsalva. Remaining subjects were categorized as having normal diastolic function.

Finally, 158 Caucasian volunteers [60 subjects without diabetes and 98 patients with type 2 diabetes, mean age ± standard deviation (SD) 56.24 ± 9.78 years] were included (convenient sample). Echocardiograms allowed the division of the study population in 4 groups: **Group A**: 42 healthy controls, **Group B**: 18 subjects without diabetes with LVDD, **Group C**: 48 patients with type 2 diabetes without LVDD, **Group D**: 50 patients with type 2 diabetes & LVDD.

Height and weight were measured in all subjects to calculate the Body Mass Index (BMI in Kg/m^2^). Fasting blood samples were analyzed for: fasting plasma glucose (FPG), urea & creatinine [in order to estimate glomerular filtration rate (eGFR) according to the Cockroft-Gault formula], total Cholesterol, high density lipoproteins (HDL), low density lipoproteins (LDL), triglycerides, BNP, hs-CRP, HbA1c and fibrinogen, using the standard procedures of the biochemistry laboratory of our hospital.

The serum of the participants was kept at -70°C and was processed within 6 months after sampling using the Presage^® ^ST2 ELISA kit (Critical Diagnostics, U.S.A.) in order to measure the Soluble ST2 levels.

Statistical analysis was performed with Kruskal-Wallis test & Mann-Whitney test (for continuous variables) and x^2 ^test or Fischer exact test (for discrete variables). Spearman coefficient was used for the univariate analysis, whereas the step-wise backward method for the multivariate analysis. The main study hypothesis was that sST2 levels differed significantly among the 4 study groups (H1).

## Results

Age, sex and duration of diabetes did not differ significantly among the study groups. Statistical variability was only observed for mean BMI among the study groups (table 1) with group A to present lower mean BMI values compared with groups B (p = 0,001) and D (p = 0,002). Univariate analysis, though, in groups C and D (patients with type 2 diabetes) did not prove that BMI impact on the circulating sST2 levels. The subjects of groups B & D (LVDD present) had more frequently history of hypertension (for B: 66.7% & D: 76%) in relation to the remaining 2 groups (LVDD absent). Additionally all members of groups B and D exhibited the impaired relaxation pattern (grade 1 LVDD) in the echocardiograms.

**Table 1:** Demographic characteristics of the study population

VARIABLE	GROUP	Mean	**S.D**.	p-value
**AGE** **(years)**	A	55,10	10,50	0,107
		
	B	60,33	9,65	
		
	C	54,87	9,94	
		
	D	56,98	8,80	
		
	TOTAL	56,24	9,78	

**SEX** **(% males)**	A	61,90	NA	0,081
		
	B	77,80	NA	
		
	C	45,80	NA	
		
	D	50,00	NA	
		
	TOTAL	58,90	NA	

**DURATION OF DIABETES (years)**	A	NA	NA	0,136
		
	B	NA	NA	
		
	C	2,81	2,50	
		
	D	4,97	6,03	

**BMI** **(Kg/m^2^)**	A	27,83	4,62	**0,005**
		
	B	32,63	6,45	
		
	C	29,41	5,13	
		
	D	31,69	7,77	

We used the Kruskal-Wallis test to analyze the variability of age, duration of diabetes & BMI and the chi squared test to analyze the sex variability between the 4 study groups. P-value found significant only for BMI, where the relative value of group A was significantly lesser than the correspondent of group B (p = 0,001) and group D (p = 0,002). All the other possible comparisons of BMI were found to be statistically insignificant. (NA = Not applicable)

Significant variability among the 4 study groups was observed for the mean values of sST2 (p < 0.001), hs-CRP (p = 0.039), FPG (p < 0.001), total Cholesterol (p < 0.001), HDL (p < 0.001), LDL (p < 0.001), triglycerides (p = 0.002) and HbA1c (p < 0.001). Mean HbA1c of Group A was 6,08%, Group B 6,46%, Group C 8,67% and Group D 7,92%. Mean HbA1c of non-diabetic population was significantly lower than the correspondent of patients with type 2 diabetes, as expected. Mean HbA1c of Group C did not differ statistically compared to the correspondent of Group D (p = 0,157). No variability was found for the mean values of BNP (p = 0.301), fibrinogen (p = 0.302) and eGFR (p = 0.393) (table 2).

**Table 2:** Results of the study's parameters

VARIABLE	GROUP	Mean	**S.D**.	p-value
**Soluble ST2 (ng/ml)**	A	9,16	4,56	**<0,001**
		
	B	9,93	3,57	
		
	C	11,31	3,05	
		
	D	14,97	5,23	

**BNP (pg/ml)**	A	28,78	13,73	0,301
		
	B	33,37	19,88	
		
	C	40,06	31,39	
		
	D	29,63	22,55	

**hs-CRP** **(mg/lt)**	A	3,04	4,45	**0,039**
		
	B	3,34	4,87	
		
	C	5,61	6,21	
		
	D	6,35	5,79	

**Fibrinogen** **(gr/lt)**	A	4,00	0,81	0,302
		
	B	3,84	0,60	
		
	C	4,35	1,55	
		
	D	3,82	1,21	

**Fasting Plasma Glucose** **(mg/dl)**	A	94,71	10,24	**<0,001**
		
	B	100,00	11,48	
		
	C	137,75	37,59	
		
	D	136,54	30,05	

**eGFR** **(mlt/min)**	A	106,70	27,05	0,393
		
	B	107,44	27,90	
		
	C	120,58	44,68	
		
	D	124,50	43,61	

**Total Cholesterol (mg/dl)**	A	211,57	34,83	**<0,001**
		
	B	222,00	44,74	
		
	C	208,88	44,95	
		
	D	173,64	39,03	

**HDL** **(mg/dl)**	A	51,24	11,67	**<0,001**
		
	B	49,33	14,42	
		
	C	47,25	15,97	
		
	D	39,60	9,96	

**LDL** **(mg/dl)**	A	136,83	30,12	**<0,001**
		
	B	143,84	36,66	
		
	C	137,13	37,22	
		
	D	101,22	36,66	

**Triglycerides (mg/dl)**	A	116,52	43,80	**0,002**
		
	B	148,56	47,83	
		
	C	124,25	52,77	
		
	D	159,30	73,90	

**HbA1c** **(%)**	A	6,08	0,36	**<0,001**
		
	B	6,46	0,38	
		
	C	8,67	2,08	
		
	D	7,92	1,21	

We used the Kruskal-Wallis test to analyze the variability of the continuous variables of the study. Significant variability among the 4 study groups was noted for the mean values of sST2, hs-CRP, FPG, total Cholesterol, HDL, LDL, Triglycerides and HbA1c. No variability was found for the mean values of BNP, fibrinogen and eGFR.

**Figure 1:**
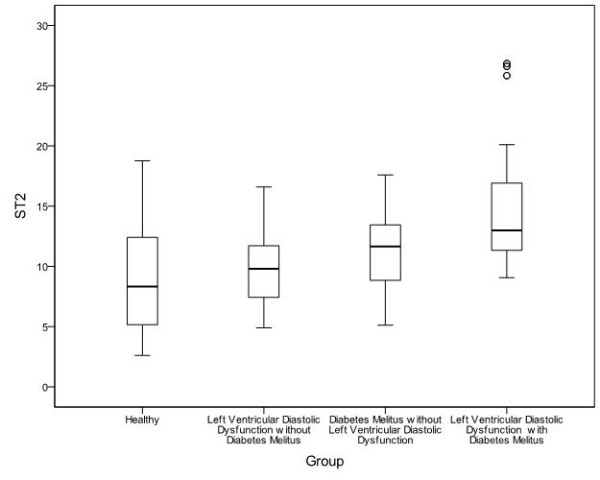
**Mean sST2 value for each study group**. Patients with type 2 diabetes and left ventricular diastolic dysfunction have the highest mean sST2 (14.97 ± 5.23 ng/ml) among the 4 study groups, even higher than the correspondent of 11.31 ± 3.05 ng/ml of patients with type 2 diabetes without left ventricular diastolic dysfunction (p = 0.001). Furthermore, both 14.97 ± 5.23 ng/ml & 11.31 ± 3.05 ng/ml are higher than the correspondent of those subjects without diabetes. Mean sST2 at 9.16 ± 4.56 ng/ml of healthy controls does not statistically differ compared to the correspondent of 9.93 ± 3.57 ng/ml of subjects without diabetes with left ventricular diastolic dysfunction (p = 0.514).

**Soluble ST2 **(mean ± SD) was 9.16 ± 4.56 ng/ml for healthy controls (Group A), 9.93 ± 3.57 ng/ml for subjects without diabetes with LVDD (Group B), 11.31 ± 3.05 ng/ml for patients with type 2 diabetes without LVDD (Group C) and 14.97 ± 5.23 ng/ml for patients with type 2 diabetes & LVDD (Group D) (Figure 1). Soluble ST2 levels did not differ significantly between groups A and B (subjects without diabetes) (p = 0.514). Statistically significant difference was observed between healthy controls (group A) and patients with type 2 diabetes without LVDD (group C) (p = 0.007). Moreover, patients with type 2 diabetes with LVDD (group D) had the highest serum levels of sST2 among the 4 study groups (table 3).

**Table 3:** Comparison of mean value of sST2, BNP and hs-CRP between the 4 study groups

Comparison	p-value		
	
	sST2	BNP	hs-CRP
A vs B	0,514	0,628	0,872

A vs C	**0,007**	0,213	**0,004**

A vs D	**<0,001**	0,207	**0,017**

B vs C	0,121	0,863	**0,041**

B vs D	**<0,001**	0,504	**0,048**

C vs D	**0,001**	0,091	0,659

**Overall**	**<0,001**	0,301	**0,039**

Soluble ST2 was significantly different between group A (healthy controls) and patients with type 2 diabetes without (group C) LVDD (p = 0.007). Moreover, patients with type 2 diabetes with LVDD (group D) had the highest mean serum level of sST2 compared to the correspondent of the rest 3 subject groups: group A (p < 0.001), group B (p < 0.001) and group C (p = 0.001). The respective mean values of BNP for the 4 study groups could not achieve statistical difference. All subjects with type 2 diabetes presented higher hs-CRP levels compared to the non-diabetic population of the study.

The respective mean values of **BNP **for the 4 study groups were: A: 28.78 ± 13.73 pg/ml, B: 33.37 ± 19.88 pg/ml, C: 40.06 ± 31.39 pg/ml and D: 29.63 ± 22.55 pg/ml without statistically significant differences between the 4 study groups (table 3).

The mean **hs-CRP **of group A was 3,04 ± 4,44 mg/l, group B 3,34 ± 4,87 mg/l, group C 5,61 ± 6,21 mg/l and group D 6,35 ± 7,79 mg/l. No difference was found for the mean values of hs-CRP between groups A *vs *B and C *vs *D. All subjects with type 2 diabetes (Groups C and D) presented higher hs-CRP levels compared to the non-diabetic population (Groups A and B) of the study (table 3).

When focusing in **Group C **(patients with type 2 diabetes without LVDD), univariate analysis showed that sST2 levels correlated positively with hs-CRP levels (p = 0.04), fibrinogen (p = 0.027), fasting plasma glucose (FPG) (p < 0.01), HbA1c (p < 0.01) and negatively correlated with the HDL (p < 0.01), sex (females) (p < 0.01), history of hypertension (p = 0.045) and chronic obstructive pulmonary disease (p = 0.01). No correlation was confirmed between sST2 and age (p = 0.513), BMI (p = 0.603), duration of diabetes (p = 0.297), BNP (p = 0.512), eGFR (p = 0.070), total Cholesterol (p = 0.580), LDL (p = 0.613), Triglycerides (p = 0.091). Multivariate analysis manifested HDL, HbA1c, sex, history of hypertension and COPD as the most powerful and independent parameters of sST2, that predict and/or interpret the 81% of sST2 variability in this group.

Similar statistical analysis in **Group D **(patients with type 2 diabetes & LVDD) revealed that sST2 levels positively correlated with HbA1c (p < 0,01) and hs-CRP levels (p = 0,042) and negatively only with HDL (p = 0.041). Multivariate analysis, though, manifested solely HbA1c as the unique independent parameter that affects the serum ST2 value in group D.

## Discussion

The results of the present study indicate that type 2 diabetes (with or without LVDD) is connected with higher sST2 levels that positively correlate with hs-CRP levels and the extend of glycemic control (ΗbA1c).

Today it is known that type 2 diabetes is associated with low-grade chronic inflammation that in part emanates from activation of the innate immune system [22] that gives rise to the acute phase response in which CRP plays a fundamental role [22] and several studies have documented the connection between high CRP levels and type 2 diabetes in the presence or absence of obesity [23,24]. Soluble ST2 levels have also been found elevated in numerous inflammatory disorders which are thought to exert a protective effect and from this point of view the correlation of sST2 levels with CRP levels in diabetics in our study most possibly reflects the inflammatory component of diabetes. In this context, increased sST2 levels represent a counterregulatory mechanism to chronic inflammation. In accordance with the above speculation is also the observed correlation between sST2 levels and extent of glycemic control as indicated by HbA1c levels.

Patients with type 2 diabetes and grade 1 LVDD, an early finding of diabetic cardiomyopathy [25,26], exhibited the highest sST2 levels in our study. The lack of diabetic patients with higher grades of LVDD did not allow further remarks on the quantitative relationship between sST2 levels and the grade of LVDD in the present study and further research is needed at this point. The increased sST2 levels in patients with diabetes and LVDD cannot be attributed to chronic inflammation or increased glycemic burden since no statistically significant differences for hs- CRP and HbA1c have been observed between the two groups of diabetic patients.

Several studies have established the prognostic role of sST2 levels in myocardial infarction, heart failure, right ventricle function and dyspneic states, while there are no available data about IL-33 levels in cardiovascular disorders [13]. Today studies from animal models suggest that sST2 is more than just a marker in cardiovascular disease and implicate IL33/ST2 signaling as an important protective pathway in which the role of sST2 remains obscure [13]. In states of oxidative stress as in diabetes increased expression of endothelin 1 has been found paradoxically to restore diastolic dysfunction [27] and endothelin 1 is a known inducer of sST2 expression and inhibitor of IL-33 signaling through p38 MAPK [28]. From this point of view, increased levels of sST2 in patients with diabetes and LVDD might be an epiphenomenon of other counteregulatory mechanisms such as endothelin 1 or to signify patients with failure of IL-33-induced cardioprotection, through sST2 acting as a decoy receptor.

TLRs are broadly distributed on immune cells and represent primordial recognition receptors mediating activation of the innate immune system [29]. Several recent studies have revealed attenuation of TLR4 and TLR2 signaling by the ST2 pathway with subsequent diminished production of proinflammatory cytokines [30-32]. In relation to infectious disease administration of sST2 protects mice against endotoxin-induced shock [31] and the IL-33/ST2 signaling pathway also prevents an inappropriate parasite specific Th1 polarized response by induction of IL-4, IL-9 and IL-13 [33] and the above findings suggest a major regulatory role of the ST2 pathway in controlling aberrant inflammatory response.

IL-33/ST2 signaling is also implicated in autoimmune disease and in an experimental animal model of diabetes I induction by multiple doses of streptozotocin this pathway seems to exert a protective effect possible through balancing Th1/Th2 response [34]. Additionally endothelial cells from type I diabetic mice have been found to exhibit enhanced inflammatory responses after stimulation of TLR2 and TLR 4 through augmented NF-kB activation[35].

The role of IL-33/ST2 pathway in type I and II diabetes remains vastly unknown today. In type II diabetes the molecular mechanisms that induce inflammatory mediators are still being elucidated and TLRs are considered primary effector molecules in this process that are also activated by nutritional factors as FFA. Ligand binding by TLRs triggers the inflammatory cascade and insulin homeostasis dysregulation [22]. Apart from the innate immune system the adaptive immunity seems also to be implicated in the chronic inflammation of type II diabetes and CD4-positive T lymphocytes are the first cells to infiltrate fat tissue [36]. From this point of view IL-33/ST2 pathway might prove to be a crucial pathway in balancing the intensity of inflammation in diabetes.

Subjects without diabetes ± LVDD exhibited similar mean serum ST2 values and this underlines the fact that sST2 does not represent a mere index of diastolic dysfunction but rather an indicator of a deranged metabolic environment of chronic inflammation and oxidative stress in which cardiovascular damage develops.

In conclusion, this is the first study to document elevated sST2 levels in type 2 diabetes. Chronic inflammation most possibly establishes the basis of increased soluble ST2 levels and diastolic dysfunction seems also to be connected with further increase of this biomarker with still obscure mechanisms.

## Conclusion

This is the first study to document increased levels of soluble ST2 in patients with type 2 diabetes in general with higher elevations in the presence of LVDD. Further research with bigger populations needs to be done in order to abut on more general conclusions.

## Abbreviations

LVDD: Left ventricular diastolic dysfunction; eGFR: Estimated glomerular filtration rate; hs-CRP: High sensitivity C-reactive protein; BNP: B-type natriuretic peptide; HbA1c: Glycosylated hemoglobin; FPG: Fasting plasma glucose; HDL: High density lipoproteins; LDL: Low density lipoproteins; SD: Standard Deviation.

## Competing interests

The authors declare that they have no competing interests.

## Authors' contributions

EF, AM, KT, EB, TE, GD and SR have made substantial contributions to conception and design of the study, acquisition of data, analysis and interpretation of data. GP performed the statistical analysis of the study. SF, ATG and ST have made substantial contributions to the performance of all the echocardiographies. SM participated in the measure of soluble ST2. All authors read and approved the final manuscript.
